# Catalytically Active Amyloids as Future Bionanomaterials

**DOI:** 10.3390/nano12213802

**Published:** 2022-10-28

**Authors:** Rodrigo Diaz-Espinoza

**Affiliations:** Departamento de Biología, Facultad de Química y Biología, Universidad de Santiago de Chile, Santiago 3363, Chile; rodrigo.diaz.e@usach.cl

**Keywords:** peptides, amyloids, catalysis, nanomaterials, cofactors, enzymes, esterase, phosphoesterase, redox

## Abstract

Peptides and proteins can aggregate into highly ordered and structured conformations called amyloids. These supramolecular structures generally have convergent features, such as the formation of intermolecular beta sheets, that lead to fibrillary architectures. The resulting fibrils have unique mechanical properties that can be exploited to develop novel nanomaterials. In recent years, sequences of small peptides have been rationally designed to self-assemble into amyloids that catalyze several chemical reactions. These amyloids exhibit reactive surfaces that can mimic the active sites of enzymes. In this review, I provide a state-of-the-art summary of the development of catalytically active amyloids. I will focus especially on catalytic activities mediated by hydrolysis, which are the most studied examples to date, as well as novel types of recently reported activities that promise to expand the possible repertoires. The combination of mechanical properties with catalytic activity in an amyloid scaffold has great potential for the development of future bionanomaterials aimed at specific applications.

## 1. The Amyloid State

Amyloids were first described as pathological deposits associated with the abnormal accumulation of sugars in several tissues such as diseased brains [1]. Their proteinaceous nature was later recognized but their structural details remained unexplored for decades [1,2]. Early attempts at characterization using X-ray diffraction of aggregated globulins provided evidence of a structural architecture characterized by intermolecular beta sheets that extended bidirectionally perpendicular to the direction of the strands [2,3]. This cross-b motif was then experimentally confirmed to be a hallmark in the structure of most amyloids [4,5]. Amyloids have since been recognized as a pathological conformation accessible to diverse proteins in many human diseases, including Alzheimer’s (AD) and Parkinson’s diseases [6,7,8]. However, several landmark experiments demonstrated that the amyloid state is not restricted to proteins involved in pathological conditions but can also be accessible to virtually any protein under the appropriate experimental conditions [6,8,9,10,11,12]. The resulting fibrils can in some cases lead to a novel pathological condition [13]. However, at the same time, the list of proteins and peptides that are naturally found in the amyloid state fulfilling a specific physiological role has exponentially increased in recent years [8,14,15,16]. These functional amyloids possess unique features that have been exploited by nature to provide a wide variety of physiological adaptations, such as mechanical support in bacterial biofilms, information exchange through the bacterial pili, and hydrophobic coating, among many others. The seeming universality of the amyloid fold is additionally supported by the discovery that small peptides can also self-assemble into amyloids that exhibit their archetypal structural features [17,18,19]. These peptides typically include highly hydrophobic sequences extracted from pathological proteins or larger peptides [20]. Hydrophobicity plays an important role in excluding water molecules and thus forming a nonpolar core that complements the stability provided by the hydrogen-bonded beta sheets [3,6,21]. However, polar peptides can also form amyloids when hydrophobic groups are intercalated in the sequence, and/or the polar sidechains provide additional hydrogen bonds [22].

**Figure 1:**
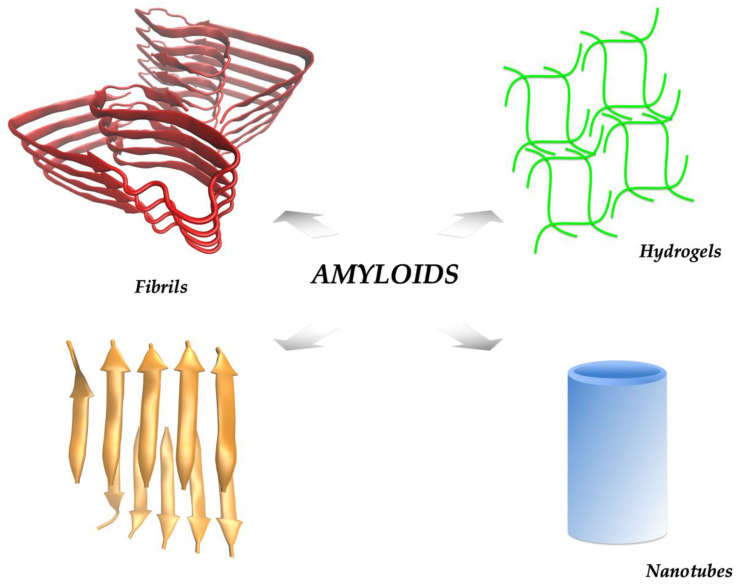
The amyloid state can be reached by proteins and peptides, which according to their sequences and particular experimental conditions can self-assemble into different supramolecular arrangements. For the amyloid fibrils, colored arrows indicate beta sheets. A full-length tridimensional structure of Ab42 amyloids (in red, pdb code 5OQV) shows the typically more complex architectures of amyloids formed by larger peptides and proteins [38]. The amyloid state of a catalytically active small peptide (SDIDVFI) is shown for comparison in orange [39].

For both peptides and proteins, the amyloid state is a highly stable conformation capable of withstanding harsh physical and chemical conditions [12,23]. Such stability emerges from a combination of interactions that include an extensive network of hydrogen bonds in the intermolecular beta sheet, secondary contacts among sidechains in the form of hydrogen bonds, ionic bridges, hydrophobic interactions, and stacking interactions among aromatic groups [3]. The amyloid state of a protein is thermodynamically more stable than its corresponding native state, which becomes a critical (and sometimes irreversible) factor in the clearance of amyloid deposits in diseased tissues [9,24]. However, this high stability has been recognized as an opportunity for the design of novel bionanomaterials based on amyloid scaffolds. For instance, peptides with relatively soluble sequences can self-assemble into amyloids that form hydrogels, a soluble amyloid state (Figure 1) [25,26,27]. These hydrogels can then serve as scaffolds to grow cells and form organoids or tissues in vitro, where they can also trap specific growth factors and promote selective cell proliferation and differentiation [28]. Amyloids can also form nanotubes, which can be further functionalized for diverse applications [29,30,31,32]. The amyloid surface can also interact with specific components present in the solution. Many amyloids bind metals with high affinity [19,33,34,35]. Hence, amyloids can be used for the decontamination of heavy metals from polluted water [36,37].

## 2. Catalytic Amyloids

The value of amyloids as future bionanomaterials was significantly boosted when the first catalytically active amyloids were reported [40]. Using the active site of carbonic anhydrase as a template, Ruffo et al. designed seven-residue peptides with sequences containing alternating histidines with hydrophobic residues. Intercalation of hydrophobic and polar groups is a strategy to design amyloids that exploits the intrinsic structural alternation of residues in a beta sheet, from which a nonpolar core can emerge while the polar residues are exposed to water [41]. The solvent-accessible amyloid surface can then become filled with potentially reactive groups. The resulting amyloid fibers can bind zinc ions through the exposed histidine and recreate the active site of carbonic anhydrase, giving rise to a bioinspired bionanomaterial. The highly ordered amyloid structure provides repetitive active sites along the surface, which can promote bidimensional catalysis [42]. The bioinspired catalyst thus forms by spontaneous self-assembly of the small peptide into an amyloid state. The hydrophobicity of the nonpolar groups correlated directly with the catalytic activity. Moreover, adding glutamine in the sequence yielded the most active peptide (IHIHIQI). Using similar strategies, different catalytic amyloids have been reported (Table 1).

## 3. Peroxidase-like Activity

### 3.1. Esterase Activities

**Figure 2:**
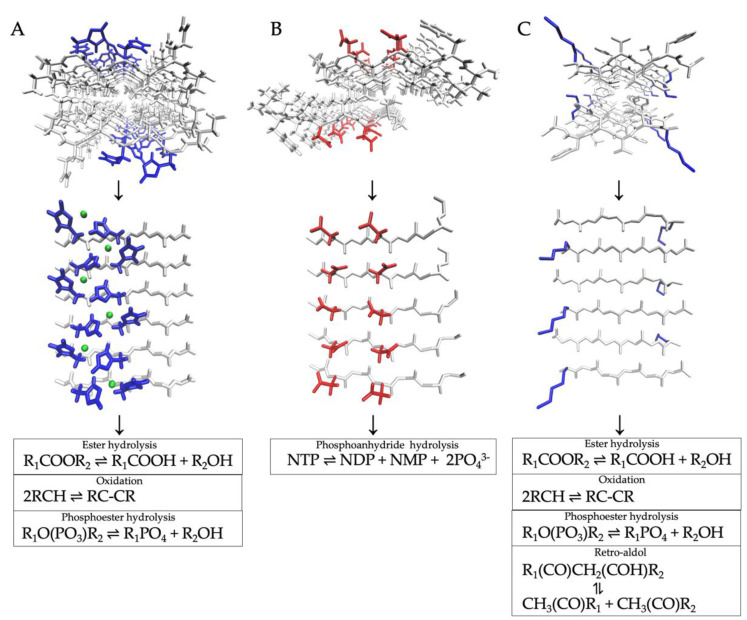
Three-dimensional structures of representative catalytic amyloids along with their catalyzed reactions. The amyloid assembly is shown in full-atomic front view and from a surface perspective in which the catalytically active and non-active residues are highlighted in blue/red and white, respectively. (**A**) Structure of peptide IHIHIQI in the amyloid state obtained by NMR and computational approaches (pdb code 5UGK) [43]. Catalytic histidines are highlighted in blue in coordination bonding with Zn^2+^ ions (in green). (**B**) A computational model of self-assembled peptide SDIDVFI is shown with its active aspartate residues (in red) [39]. (**C**) Crystal structure of an amyloid formed by peptide KLVFFA obtained by X-ray diffraction and NMR analyses [45]. Peptides derived from this sequence can self-assemble into amyloids with different catalytic activities, in which lysine (in blue) or similarly basic residues are essential for activity.

Most catalytic amyloids reported to date exhibit hydrolytic activities. Esterase-like activity has been the most studied case thus far. The activity is typically tested using the hydrolysis of the ester bond of the model compound para-nitrophenyl acetate (pNPA). This activity can emerge from different combinations of histidine and nonpolar residues. For instance, the amyloid-mediated esterase activity of peptide IHIHIQI is maintained if isoleucine is replaced by valine or leucine, but the values for the kinetic constants (*k_cat_* and *k_cat_*/*K_M_*) are in general reduced [40]. Two histidines in the peptide sequence appear to be the sweet spot for optimal catalytic activity. Simulations and NMR experiments showed that three to four zinc ions are coordinated to three nearby histidine residues on the amyloid surface, which seems to recapitulate the three-dimensional arrangement of histidine residues on the active sites of carbonic anhydrase (Figure 2A) [43]. The intermolecular beta-sheets can arrange in a parallel or antiparallel fashion depending on the specific sequence, which has a direct impact on the level of esterase activity. Moreover, specific supramolecular arrangements such as the twisting of fibrils can also affect activity, by allowing better exposure to substrates and providing at the same time the necessary flexibility for catalysis. The type of non-catalytic solvent-exposed residue such as glutamine appears to have an essential role in driving the specific final architecture of the amyloid and hence its activity, probably due to the secondary contacts mentioned above. In any case, esterase activity is exclusive of the amyloid state and not the unassembled peptide or other alternative aggregated states.

A recent work showed the potential role of peptide IHIHIQI in the amyloid state as a bioactive nanomaterial with esterase activity [44]. The amyloids were active against not only pNPA but also other para-nitrophenyl esters. Several self-assembled peptide variants (peptides IHIHIYI and IHVHLQI) also exhibited a similar esterase activity. Interestingly, the amyloids were then tested as biocatalysts of the degradation of a model plasticizer of environmental concern due to its potential leakage from industrial applications. All the amyloids caused a Zn-dependent partial elimination of the molecule, which reached 80% degradation at 50 °C with peptide IHVHLQI.

Amyloids with esterase activity could also be obtained using phenylalanine as a single amino acid [46]. Interestingly, these amyloids are formed by less conventional cross-beta sheets in which the free N- and C-termini form hydrogen-bonded beta strands and coordinate zinc ions, while the phenyl moiety engages in parallel stacking interactions in a nonpolar pocket that further stabilizes the overall structure. Though no peptide bonds were present, the activity resembled that obtained with peptide IHIHIQI in terms of kinetic parameters, strongly suggesting that the catalytic power of amyloids is not restricted to specific residues, sequences, or bonds, and it seems to mainly rely on reducing the reaction space to a bi-dimensional surface space [42,47]. Adding to that, amyloid-mediated esterase activity can even be obtained in the absence of zinc ions or any other metal ion. Peptide HSGQQKFQFQFEQQ for instance self-assembles into amyloids with metal-independent esterase activity [48]. Although the associated activity was much lower than that of other amyloids, it still shows that catalysis can emerge solely with histidine residues orderly exposed along the amyloid surface. Alternating histidine with tyrosine residues led to peptides (HYHYHYH, YHYHYHYH, and HYHYHYHYH) self-assembled into amyloids that also showed metal-independent esterase activity [49]. The activities of these amyloids (HY) yielded values within the range of those obtained with peptide HSGQQKFQFQFEQQ. Peptides that combine histidine with hydrophobic D-amino acids in their sequence (Peptide HF^D^F^D^ and later peptide HL^D^LIHL^D^L) can also self-assemble into catalytic amyloids with Zn-independent esterase activity similar to that obtained with the above amyloids [50,51,52]. In these cases, the amyloids formed catalytically active hydrogels.

Interestingly, amyloids formed in vitro with a synthetic form of the pathological peptide involved in Alzheimer’s disease (Ab42) were recently shown to catalyze the hydrolysis of pNPA in absence of metal ions [53]. The esterase activity was similar to that obtained with metal-independent catalytic amyloids. Moreover, adding metal ions to the amyloids has either no impact or an apparent inhibitory effect on the activity. The Ab42 amyloids also showed hydrolytic esterase activity against a thiol surrogate of acetylcholine, an important neurotransmitter, which can lead to potentially novel pathological pathways. The same group later showed that amyloids formed with the hormone glucagon, which are also involved in pathology, exhibit esterase activity in vitro using pNPA as the model substrate and without the need for metal ions [54]. The resulting catalytic activities were similar in range to that obtained with Ab42. Surprisingly, these amyloids also showed hydrolytic activity against an analog of a physiologically relevant acid fat (para nitrophenyl palmitate), which was effectively cleaved at the ester bond joining the acid fat with the chromogenic group. The enzyme-catalyzed hydrolysis of fats in the food industry is widely used and has diverse applications such as food processing, removal of waste fats, etc. [55,56]. Although glucagon is rather a large peptide and thus it may not be optimal as a bioactive nanomaterial for such purposes, amyloids formed by smaller peptides either derived from glucagon or those used in the aforementioned studies could become attractive alternatives to explore their potential as industrial biocatalysts.

### 3.2. Phosphoesterase Activities

Amyloid-mediated catalysis of other hydrolytic activities has also been reported. A small modification of peptide IHIHIQI (peptide IHIHIYI) produced amyloids that catalyzed the copper-dependent hydrolysis of the phosphoester bond of paraoxon, a widely used pesticide [57]. Interestingly, amyloids forming with the same peptide also exhibit zinc-dependent esterase activity, suggesting that at least in this case the amyloid serves as a surface that can switch activities by simply exchanging the metal ion [44,58]. Paraxon and similar industrial pollutants can thus become future targets for amyloid nanomaterials.

Another type of hydrolytic activity is the catalysis of the hydrolysis of phosphoanhydride bonds such as the one present in adenosine triphosphate (ATP). My group showed that a peptide derived from the highly conserved active site of RNA polymerases (NADFDGDQMAVHV) self-assembled into amyloids that hydrolyzed ATP into adenosine di- (ADP) and mono-phosphate (AMP) in the presence of manganese ions (Mn) [59]. In a follow-up work, we showed that a smaller peptide containing the partial sequence of the active site of a DNA polymerase (SDIDVFI) formed catalytically active amyloids that exhibited the same Mn-dependent hydrolytic activity (Figure 2B) [39]. Furthermore, all four ribonucleotides were hydrolyzed into their NDP and NMP forms as well as deoxyadenosine triphosphate (dATP) into dADP and dAMP. Interestingly, the abovementioned glucagon amyloid fibrils also exhibited hydrolytic activity towards the phosphoanhydride bonds of ATP albeit without the need for metal cofactors [54] The kinetic constants exhibited values within the range of those obtained in our works. Moreover, these amyloids were also active against the phosphoester bonds of para-Nitrophenyl Orthophosphate (pNPO) with an overall hydrolytic activity higher than that with ATP. As with the amyloid-mediated catalysis of esterase activity, the hydrolytic activity against phosphoester and phosphoanhydride bonds is exclusively ascribed to the amyloid state and not the monomer or oligomeric peptides.

### 3.3. Redox Activities

Although catalysis of hydrolytic reactions remains the most extensively studied amyloid-mediated activity to date, the repertoire of possibilities is not restricted to hydrolysis. For instance, several studies have shown that peptide Ab42 can catalyze the formation of reactive oxidative species (ROS) when bound to copper ions [60]. However, these works did not explore a correlation between the activity and the aggregated state of Ab42. In a recent study, Arad et al. showed that mature amyloid fibrils formed with Ab42 not only exhibit an esterase activity but can also catalyze the oxidation of dopamine (DA) and adrenaline (Adr), two relevant neurotransmitters [53]. DA was successively oxidized into a melanin derivative, which becomes dysregulated in AD, whereas Adr was converted to Adrenochrome.

Makhlynets et al. showed that amyloids self-assembled with peptide IHIHIQI can catalyze the oxidation of 2,6-dimethoxyphenol [61]. The catalysis was copper-dependent and very sensitive to the peptide/metal ion ratio. A similar result is observed with HY amyloids [49]. In addition to displaying esterase activity, these amyloids exhibited an electrochemical response when coupled to the oxidative polymerization of pyrrole. The activity was copper-dependent and relied on a tyrosine-mediated radical mechanism. Amyloid-catalyzed oxidation of organic compounds can have wide biotechnological implications. Indeed, two recent works showed that phenylalanine and copper can form metal-amyloid complexes that catalyzed the oxidation of a wide variety of organic compounds and could serve as future bioactive nanomaterials [62,63]. Makam et al. reported that these cooper-bound self-assembled phenylalanine amyloids caused the oxidation of 2,4-dichlorophenol (DP), with relative activities significantly higher than those obtained with laccase, a well-known enzyme that is regularly used in oxidation processes in the industry [63]. Moreover, the amyloids catalyzed the oxidation of many other phenolic molecules that mimic industrially relevant compounds. Liu et al. showed a similar result in which copper-bound phenylalanine amyloids were active against DP but also towards DA [62]. It is noteworthy that DA has been independently shown as an effective substrate for two different catalytic amyloids. Moreover, in the case of Ab42 this activity did not require copper or any other metal.

In another recent study, oligohistidine peptides of different lengths (from 1 to 20 histidines) were allowed to self-assemble into intermolecular assemblies composed of beta sheets with the aim of mimicking several features of the active site of several peroxidases [64]. All peptide assemblies longer than two residues catalyzed the oxidation of 3,3′,5,5′-tetramethylbenzidine by hydrogen peroxide (H_2_O_2_). Although no experiments were conducted to confirm the amyloid nature of these assemblies, the structural data and simulations showed that the architecture of the assemblies is highly reminiscent of an amyloid conformation. Interestingly, the most active peptide (15 residues long) endured many cycles of drastic temperature and pH changes, supporting again the high stability of these bioactive nanomaterials. Peroxidase-like activity was also reported with self-assembled amyloids formed with peptide LALHLFL, a derivative of peptide IHIHIQI that was previously shown to bind hemin and form hemin-amyloid assemblies that catalyzed the cyclopropanation of 4-(trifluoromethyl)styrene with ethyl diazoacetate [65,66]. The amyloids catalyzed the oxidation of 3-ethylbenzthiazoline-6-sulfonate (ABTS) and 3,3′,5,5′-tetramethylbenzidine (TMB) [65]. Replacing alanine with other hydrophobic residues yielded active self-assembled peptides such as peptide LMLHLFL, the most active tested sequence. All these redox activities are again exclusively tied to the amyloid state but the structural characterization of the active site at high resolution is still lacking. Future experiments should shed light on the mechanistic aspects of amyloid-mediated catalysis of redox reactions in with the aim of designing future amyloid nanomaterials.

### 3.4. Catalytic Amyloids Derived from Ab42

Small peptides containing sequences from Ab42 can also self-assemble into catalytically active amyloids. Metha et al. designed a peptide from a sequence derived from the hydrophobic core of Ab42 (KLVFFAE) in which glutamate is replaced with leucine (peptide KLVFFAL) [47]. The peptide self-assembled into amyloid-like nanotubes that can catalyze a retro-aldol reaction without the need for a metal cofactor (Figure 2C). Replacing lysine with histidine (peptide HLVFFAL) resulted in self-assembled amyloids that bind hemin [67]. The hemin-amyloid assembly showed H_2_O_2_-mediated peroxidase activity that oxidized 2-methoxy phenol. The assembly was then used to catalyze the tandem reactions of hydrolysis and subsequent oxidation of 2-methoxy phenyl acetate and 2-methoxy phenol respectively, yielding a complex aromatic product.

Interestingly, covalent modification of these Ab42-derived peptides can yield catalytic amyloids with different activities. Condensation of an imidazoleacetic acid moiety to lysine in KLVFFAL (Im-KLVFFAL) resulted in self-assembled amyloid nanotubes that exhibited metal-independent esterase activity using in this case keto ester 4- nitrophenyl 4-oxopentanoate and 6-hydroxy-2-naphthaldehyde as ester substrates [68]. In another work, the same sequence (KLVFFAL) was used to synthesize covalent lipid-peptide assemblies [69]. An inverted derivate sequence (FFVK) was covalently attached to decanoic acid at the N-terminal (C_10_-FFVK). Long-term incubation of this modified peptide led to ordered nanotubes highly reminiscent of amyloids. The nanotubes catalyzed the retro-aldol condensation between an aromatic alcohol (methodol) and 6-methoxy-2-naphthaldehyde with high substrate selectivity. The catalysis of a carbon-carbon bond by amyloid-like assemblies formed with a small-size peptide linked to a structurally simple lipid may have relevance in the context of ancestral enzymes. Similar retro-aldol condensations were reported using peptides RFRFRFRF and PRFRFRFRF [70]. Amyloid-like aggregates formed with these peptides catalyzed the condensation between cyclohexanone and p-nitrobenzaldehyde. Catalysis of the retro-aldol reaction was also reported for a self-assembled hydrogel formed by a peptide that combined phenylalanine with glutamate. Although the catalytic conformation of the peptide exhibited aggregated beta sheet layers, their amyloid nature was not confirmed [71].

Another peptide derivative of KLVFFAL that replaced lysine and leucine with arginine and histidine respectively (peptide RLVFFAH) self-assembled into amyloid-like nanotubes that catalyzed the hydrolysis of different phosphoester bonds that partially mimic the activity of canonical enzymes such as RNase, DNase, and phosphatase [72]. The assemblies exhibited cofactor-independent hydrolytic activity towards the RNA-like compound hydroxypropyl-4-nitrophenylphosphate, the DNA-like substrate Bis (4-nitrophenyl)phosphate, and pNPO as a substrate surrogate of phosphatase. Ab42-derived peptide KLVFFAE has also served to design amyloid-like scaffolds onto which enzymes can be adsorbed. Chatterjee et al. recently reported that a small modification of this sequence (peptide RLVFFAL) formed amyloid-like nanotubes that bind alcohol dehydrogenase (ADH) [73]. ADH remained active on the amyloid surface and was used as a fluorescent reporter upon the catalytic oxidation of aromatic alcohols to naphthaldehyde, a fluorescent product. Subsequent binding of sarcosine oxidase and catalase yielded an amyloid-enzyme network that triggered the catalysis of cascade reactions, which could be followed by fluorescence. A similar hybrid strategy was used to colocalize sarcosine oxidase and hemin onto self-assembled nanotubes formed by peptide Im- KLVFFAL [74]. The resulting amyloid-enzyme-cofactor assembly exhibited convergent oxidase and peroxidase activities. These novel strategies could be used in the future to design hybrid nanomaterials with extended bioactivities. Since KLVFFAL peptide tends to self-assemble into nanotubes, most of the above activities are associated with this specific conformation. In addition to providing reactive surfaces, nanotubes may facilitate catalysis inside the tubes [47].

### 3.5. Stability of Catalytic Amyloids

A future implementation of catalytic amyloids as nanomaterials for in situ applications would require the characterization of not only their activities but also their stability and mechanical properties. Amyloids are considered highly stable conformations but experimental determinations of their stability in terms of free energy values is a complex task that depends on many factors [12]. For instance, if the amyloid forms by the self-assembly of a formerly-folded protein, then the unfolding of the protein towards folding intermediates or unfolded states must be evaluated in the calculation of a global free energy. Moreover, the amyloid state is typically associated to phase changes due to their hydrophobic nature, which often leads to precipitation or gel-like final states, resulting in non-equilibrium processes that are not amenable to classic thermodynamics. Still, several works have shown that thermodynamic factors such as temperature and pressure can be quantitatively used for assessing the stability of amyloids [75,76,77,78].

Due to the polymeric nature of amyloids, their mechanical properties are easily accessible through experiments and have thus been extensively studied in the literature. For instance, early studies showed that amyloids formed by the hormone insulin have strength and Young’s modulus (stiffness indicator or persistent length) comparable to those of steel [79]. However, later works showed that these mechanical indicators can vary widely among different amyloids, which highlights the pivotal role of the specific protein or peptide sequence [80,81]. The hydrogen-bonded core structure of amyloids allows for spontaneous breakage of the fibrils, which can also vary among different amyloids but also on external conditions such as pH [82,83]. The balance of strength and flexibility can prove relevant for the use of amyloids as nanomaterials, while the strong dependence of these mechanical properties on the specific protein or peptide sequence can allow for highly versatile design strategies. Still, no reports have specifically addressed the mechanical characterization of catalytic amyloids, which leaves an interesting future object of study.

Despite the lack of specific studies on the stability and mechanical properties of catalytic amyloids, these functional assemblies offer the possibility of indirectly assessing their structure by studying their activities, as has been done with proteins for decades. Three of the studies cited in this review have directly addressed the effect of harsh chemical and physical treatments on amyloid activity [23,62,64]. Friedmann et al. showed that the esterase activity of amyloids formed by small histidine-containing peptides is barely affected by a high concentration of salt (up to 4.75 M NaCl), temperature (95 °C), pH (2.2) or solvent exchange (90% ethanol or 50% dimethyl sulfoxide). Similarly, the amyloid fibrils formed by single phenylalanine retained their laccase-like activity after multiple treatments under extreme conditions that resemble potential industrial environments, demonstrating that the stability of catalytic amyloids is perfectly compatible with their use as nanomaterials in biotechnology. Moreover, these are to date the only reported examples in which the activity of the catalytic amyloid was compared alongside an enzyme (laccase) under different experimental conditions.

## 4. Conclusions

Since their discovery in 2014, research on catalytic amyloids has exponentially increased in recent years. Catalysis of hydrolytic reactions remains the most exploited and characterized activity to date. Peptide IHIHIQI has served as a building block to design different catalytic amyloids, while histidine has emerged as a highly versatile amino acid in the search for novel activities. The amyloid-mediated hydrolysis of ester bonds can be achieved with many different peptide sequences, which have in common the presence of intercalated histidine. These same peptides, or several derivatives, can also self-assemble into amyloids that exhibit phosphatase, oxidase, and peroxidase activities. In many cases, the simple exchange of the divalent metal decorating the amyloid surface can lead to a different catalytic activity. In enzymes, histidine also fulfills numerous roles in catalytic reactions [84]. Therefore, the design of future catalytic amyloids as future bioactive nanomaterials can benefit from the use of the active site of enzymes with active histidine groups as templates. Histidine can coordinate zinc or copper, forming highly flexible and diverse catalytic complexes. However, esterase activity can also be attained using zinc-bound self-assembled phenylalanine, which suggests that the same catalytic activity can be obtained through flexible amyloid-mediated approaches. In the case of esterase activity, metal cofactors appear to significantly enhance the observed activities compared to cofactor-independent catalytic amyloids. Further research should elucidate whether the activity of the reported cofactor-independent catalytic amyloids could be boosted by the addition of metals. For instance, the esterase activity of amyloids formed by Ab42 showed little or even a slightly detrimental effect, which may suggest that some of these amyloids may operate through different catalytic mechanisms [53]. Still, for potential industrial applications, the development of cofactor-independent catalytic amyloids may represent an attractive avenue of applied research. The recent reports on the catalytic activity of two pathological amyloids (Ab42 and glucagon) can serve as a rich source to explore novel catalytic amyloids. Pathological amyloids are typically formed by larger and much more complex sequences; hence, their sequences can be used as templates for the design of small peptides that self-assemble into catalytic amyloids. A hallmark example of this is sequence KLVFFAE, derived from Ab42. It is noteworthy that small modifications to such a small sequence can give rise to a wide repertoire of catalytic activities.

The value of catalytic amyloids as potential bioactive nanomaterials has been effectively demonstrated in several recent works. For instance, amyloid-like nanotubes forming by phenylalanine showed activities comparable to or even greater than normal enzymes, such as laccases, acting toward substrates resembling industrially relevant compounds [62,63]. Moreover, these assemblies exhibited significantly greater structural and functional resilience upon treatments under harsh conditions. The fact that a single amino acid self-assembled into an amyloid can outcompete a hallmark enzyme in several benchmarks is indicative that a rich source of catalytic activities is awaiting to be discovered. Small peptides can be engineered for better catalytic efficiency, greater selectivity, and/or improved stability. Complementary research on the mechanical and functional aspects of amyloids should prove highly beneficial to develop future amyloid-based nanomaterials with biotechnological value.

## Figures and Tables

**Table 1:** List of peptides and their specific catalytic activities in the amyloid state.

Peptide ^1,2^	Catalytic Activity	Cofactor	*k_cat_* ^2^ (s^−1^)	*k_cat_/K_M_*^2^ (M^−1^s^−1^)
Ac-IHIHIQI-Am	Ester hydrolysis ^3^	Zn	2.6 × 10^−2^	62
	Oxidation	Cu	-	-
Ac-IHIHIYI-Am	Ester hydrolysis ^3^	Zn	8.3 × 10^−3^	355
	Phosphoester hydrolysis	Cu	8 × 10^−5^	2.8 × 10^−2^
Ac-IHVHLQI-Am	Ester hydrolysis ^3^	Zn	1.76	127.7
Phe	Ester hydrolysis	Zn	-	76.5
	Oxidation	Cu	11.9	63 × 10^−3^
Ac-HSGQQKFQFQFEQQ-Am	Ester hydrolysis	None	1.95 × 10^−3^	9 × 10^−2^
Ac-HYHYHYHYH-Am	Ester hydrolysis	None	3.5 × 10^−3^	1.64
	Oxidation	Cu	-	-
HL^D^LIHL^D^L	Ester hydrolysis	None	2.8 × 10^−3^	2.9
HF^D^F^D^ ^4^	Ester hydrolysis	None	8.7 × 10^−3^	-
Ab42	Ester hydrolysis	None	1.9 × 10^−3^	0.66
	Oxidation	None	-	-
Glucagon	Ester hydrolysis ^3^	None	2.5 × 10^−3^	0.57
	Phosphoanhydride hydrolysis	None	1.6 × 10^−5^	2.7 × 10^−1^
	Phosphoester hydrolysis	None	7 × 10^−3^	59.3
Ac-NADFDGDQMAVHV-Am	Phosphoanhydride hydrolysis	Mn	2.3 × 10^−4^	4.2 × 10^−2^
Ac-SDIDVFI-Am	Phosphoanhydride hydrolysis	Mn	4.2 × 10^−6^	6.4 × 10^−2^
Ac-Oligohistidine-Am	Oxidation ^5^	None	1.3 × 10^−4^	0.7
Ac-LALHLFL-Am	Oxidation ^5^	Hemin	1.3	300
Ac-LMLHLFL-Am	Oxidation ^5^	Hemin	7.8	565
Ac-KLVFFAL-Am	Retro-aldol	None	6.2 × 10^−5^	-
Ac-HLVFFAL-Am	Oxidation ^5^	Hemin	0.24	-
Im-KLVFFAL-Am	Ester hydrolysis	None	1.5 × 10^−3^	2.1
C_10_-FFVK-Am	Retro-aldol	None	1.7 × 10−5	-
Ac-PRFRFRFRF-Am	Retro-aldol	None	-	-
Ac-RLVFFAH-Am	Phosphoester hydrolysis	None	10.9 × 10^−5^	1.58

^1^ Ac and Am denote peptides that were acetylated and amidated in their N- and C termini ends respectively. ^2^ The most active peptide with the most active substrate are shown. ^3^ Ester hydrolysis is demonstrated with different substrates. ^4^ Both unprotected and protected peptides were catalytically active. ^5^ Peroxidase-like activity is reported.
